# Retraction: A novel immunotherapy of Brucellosis in cows monitored non invasively through a specific biomarker

**DOI:** 10.1371/journal.pntd.0011002

**Published:** 2022-12-20

**Authors:** The PLOS Neglected Tropical Diseases Editors

Following publication of this article [1], concerns were raised about the reported methodology and results, availability of data and materials, and about the ethical approval procedures.

## Ethics approval

The corresponding author has provided the editors with copies of the ethical approval documentation and the application for permission for animal experiments. Editorial review noted that although the animal use application indicated a plan to study the effect of phage lysate of *Brucella* in therapy of Brucellosis, the description of procedures listed only subcutaneous injection of Brucellaphage and did not specify injection of phage-lysate of *Brucella* S19 and RB51, as carried out in [1]. The corresponding author has stated there was an inadvertent omission of phage lysate in the list of substances to be administered. It was further noted that the approved application specified 18 cattle (6 each in two test groups and 6 in a control group), while the article [1] reports the use of 21 cattle in three test groups and one control group.

## Data availability

The underlying data for this study are not included with the article; however, in post-publication follow up, the corresponding author provided the individual-level underlying data for 21 animals for all figures and tables in the article and one additional representative RT-PCR gel; the corresponding author stated that no further underlying RT-PCR gel images or microbial culture plate images are available. The corresponding author has stated that the lytic phage used in the study has been deposited at the National Centre for Veterinary Type Cultures at ICAR National Research Centre on Equines, with accession number VTCCBPA183.

### Concerns about study design and data

Concerns were raised after publication about the reliability and interpretation of some of the reported results, including that the standard deviations for the antibody titer data in Table 1 are small for biological replicates, that blood may not be a suitable tissue for study of Brucellosis, and that isolation and culture of *Brucella* is considered to have low sensitivity. In reviewing these issues, *PLOS Neglected Tropical Diseases* obtained input from two members of our Editorial Board who reviewed the underlying individual-level RT-PCR and culture results provided, which reported 100% positivity for untreated animals at all tested time points from day 0 to day 90. One consulted editor advised that culture positivity and RT-PCR positivity varies widely in Brucellosis depending on a number of factors, and that there is insufficient information reported about the potential pre-selection of animals on the basis of test positivity that may contribute to the high levels of positive results in culture and RT-PCR. A second consulted editor advised that the average sensitivity of *Brucella* isolation in culture is low and isolation of *Brucella* from all untreated infected animals at all time points is unexpected; additionally, the consulted editor advised that blood is not considered a suitable tissue for culture because *Brucella* bacteremia in cattle is very short. The corresponding author has indicated that the reported results accurately reflect what was observed and noted that RT-PCR was used to monitor levels of *Brucella*-specific RNA in the blood plasma as an indication of the presence of live *Brucella* in the body, not to demonstrate bacteremia.

PLOS has contacted the Guru Angad Dev Veterinary and Animal Sciences University. Given the cumulative advice and information received in post-publication follow-up, the *PLOS Neglected Tropical Diseases* editors consider that the concerns about the reliability of the results are not resolved. In light of concerns about differences between the reported study in [1] and the approved animal use application, and concerns about the reliability of reported results that were not resolved in post-publication follow-up, the *PLOS Neglected Tropical Diseases* Editors retract this article.

HMS and SR did not agree with the retraction and stand by the article’s findings.
